# Progress in Procalcitonin Detection Based on Immunoassay

**DOI:** 10.34133/research.0345

**Published:** 2024-04-16

**Authors:** Jiayue Huang, Yan Zu, Lexiang Zhang, Wenguo Cui

**Affiliations:** ^1^ State Key Laboratory of Targeting Oncology, National Center for International Research of Bio-targeting Theranostics, Guangxi Key Laboratory of Bio-targeting Theranostics, Collaborative Innovation Center for Targeting Tumor Diagnosis and Therapy, Guangxi Medical University, Nanning, Guangxi 530021, P.R. China.; ^2^ Oujiang Laboratory (Zhejiang Lab for Regenerative Medicine, Vision and Brain Health); Wenzhou Institute, University of Chinese Academy of Sciences, Wenzhou, Zhejiang 325000, P.R. China.; ^3^ Joint Centre of Translational Medicine, the First Affiliated Hospital of Wenzhou Medical University, Wenzhou 325035, P.R. China.; ^4^ Department of Orthopedics, Shanghai Key Laboratory for Prevention and Treatment of Bone and Joint Diseases, Shanghai Institute of Traumatology and Orthopedics,Ruijin Hospital, Shanghai Jiao Tong University School of Medicine, 197 Ruijin 2nd Road, Shanghai 200025, P.R. China.

## Abstract

Procalcitonin (PCT) serves as a crucial biomarker utilized in diverse clinical contexts, including sepsis diagnosis and emergency departments. Its applications extend to identifying pathogens, assessing infection severity, guiding drug administration, and implementing theranostic strategies. However, current clinical deployed methods cannot meet the needs for accurate or real-time quantitative monitoring of PCT. This review aims to introduce these emerging PCT immunoassay technologies, focusing on analyzing their advantages in improving detection performances, such as easy operation and high precision. The fundamental principles and characteristics of state-of-the-art methods are first introduced, including chemiluminescence, immunofluorescence, latex-enhanced turbidity, enzyme-linked immunosorbent, colloidal gold immunochromatography, and radioimmunoassay. Then, improved methods using new materials and new technologies are briefly described, for instance, the combination with responsive nanomaterials, Raman spectroscopy, and digital microfluidics. Finally, the detection performance parameters of these methods and the clinical importance of PCT detection are also discussed.

## Introduction

Procalcitonin (PCT) is a 166-amino-acid precursor produced by thyroid C cells. It is not only considered an acute indicator for differential diagnosis but also widely used as a parameter to monitor inflammatory activity [1,2]. The detection of PCT holds immense importance in the diagnosis, treatment, and prognosis evaluation of bacterial infections. By closely monitoring PCT levels, physicians can precisely identify the type and severity of the infection. This information enables them to individualize antibiotic therapy based on the specific needs of each patient, minimize antibiotic misuse, and formulate targeted treatment plans [3–5]. Marked strides have been made in the clinical immunoassay of PCT, and chemiluminescence stands out as the most widelyX employed method for PCT detection. However, this method faces challenges including an extra luminescence process, pronounced background interference, reagent instability, and suboptimal detection accuracy [6]. To address these shortcomings, numerous studies have focused on enhancing PCT immunoassays through the utilization of superior materials and advanced detection systems [7].

This article introduces recent developments in various immunoassay methods centered around PCT detection. Notably, nanomaterials have been employed as signal reporter molecules to amplify detection signals [8–10]. Although the development of nano-biological detection technology is still in its early stages of development, the superior properties of nanomaterials have brought a new dawn to rapid diagnosis. Specifically, semiconductor quantum dots (QDs) [11,12], hydrogels [13–16], and graphene [17–19] have demonstrated marked advantages in enhancing detection system performance. Furthermore, surface-enhanced Raman spectroscopy (SERS) and digital enzyme-linked immunosorbent assay (digital ELISA) also have offered distinct advantages in improving sensitivity [20,21]. The integration of emerging technologies such as nanotechnology [22,23], biochips [24], and microfluidics in immune detection clinical diagnosis [25–28] marked a new stage in PCT detection—characterized by heightened sensitivity and specificity. This convergence of technologies signifies a paradigm shift in the landscape of PCT detection toward more advanced and precise methodologies.

**Fig. 1.**
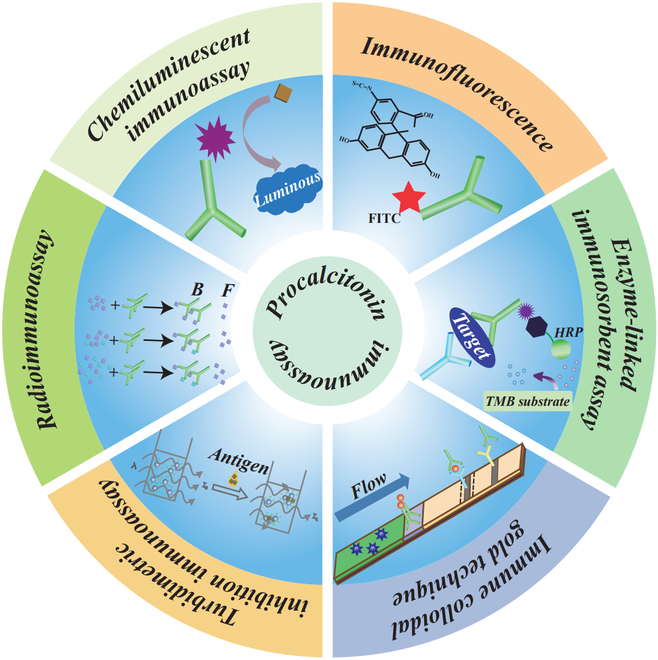
Overview of existing immunoassays for PCT detection (B: bound, F: free, FITC: fluorescein isothiocyanate, HRP: horseradish peroxidase, and TMB: 3,3′,5,5′-tetramethylbenzidine).

Therefore, this article provides a comprehensive review of the applications of various PCT immunoassay technologies in recent years, including history and recent developments. First, we briefly introduce the background and principles of several major methods such as chemiluminescence, immunofluorescence (IF), enzyme-related immunosorbent, latex-enhanced turbidity, colloidal gold immunochromatography, and radioimmunoassay (RIA) (Fig. 1), and then the detection performance and application prospects of the improved new method are introduced and evaluated. Finally, we summarized the clinical application value of PCT. We have reason to believe that this review has shed new light on the development of PCT immunoassay methods and will provide more inspiration for future research work.

## Chemiluminescent Immunoassay for PCT Detection

The application of chemiluminescence as an analytical tool dates back to the early 1950s, and since then, various branches related to this technology, such as theoretical concepts, instrumentation, and methods, have experienced important development [29–31]. Chemiluminescence, combining the specificity of antigen–antibody immune reactions with the sensitivity of chemiluminescence reactions, is well-suited for detecting various antigens, antibodies, and other biochemical analytes commonly used in clinical detection. As we know, when a substance returns from an electronically excited state to its ground state, the energy released manifests as light. Chemiluminescence labels these luminescent substances, absorbing chemical energy during reactions and releasing it as photons. In the PCT chemiluminescence immunoassay, PCT monoclonal antibodies (mAbs) are coated on a solid-phase carrier, and luminescent substances label PCT mAbs directed against another epitope, forming a double-antibody sandwich structure with a chemiluminescent substrate system. Common luminescent substances include acridinium esters [32], ruthenium terpyridin [33], luminol and its derivatives [34–36], and 3-(2′-spiroadamantyl)-4-methoxy-4-(3″-phosphoryloxy)-phenyl-1,2-dioxetane [37]. In recent years, metal nanoparticles have been utilized as catalysts, reducing agents, energy receptors, and nano-response platforms, triggering liquid-phase chemiluminescence reactions due to their special optical, catalytic, and chemical effects. This is important for chemiluminescent biodetection using metal nanoparticles as nanoprobes or nanointerfaces.

**Fig. 2.**
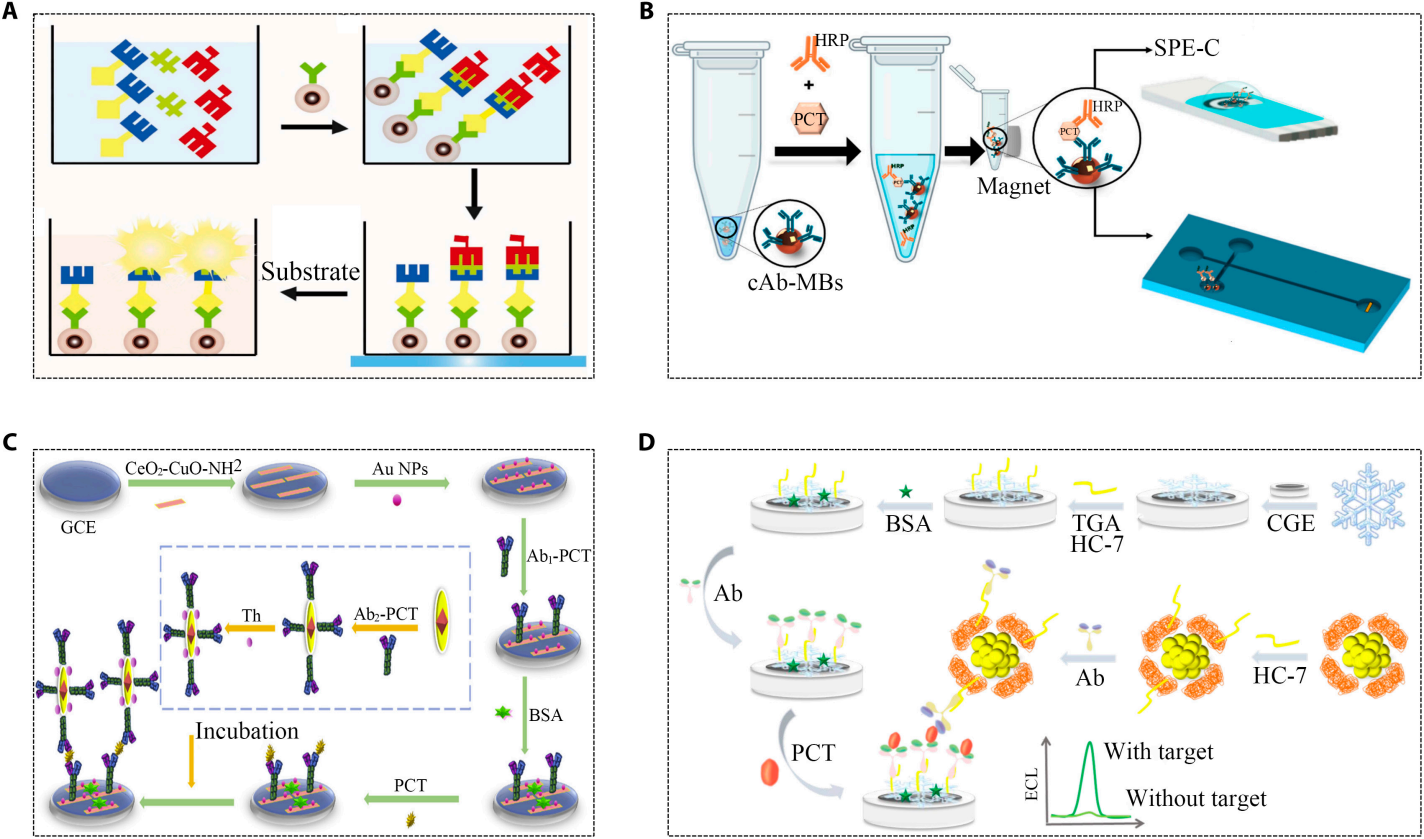
Representative demonstrations of electrochemiluminescence methods for PCT detection. (A) Schematic diagram of the proposed chemiluminescence immunoassay [38]. Copyright 2013 Japan Society for Analytical Chemistry. (B) Schematic illustration of magnetic bead-based electrochemical immunoassays SPE-C and EMC-Au [39]. Copyright 2022 MDPI. (C) Sandwich EIS detection system [40]. Copyright 2019, Elsevier. (D) Illustration of preparation of immunosensor for detection of PCT (TGA: mercaptoacetic acid, GCE: glassy carbon electrode, and HC-7: hexachloroethane zinc-heptapeptide) [41]. Copyright 2022 Elsevier.

In one study, a magnetic nanoparticle (MNP)-based assay was used to determine PCT concentration using chemiluminescent immunoassay (CLIA). This study employed N-(aminobutyl)-N-(ethylisoluminol) (ABEI) as the luminescent substrate, coupled with magnetic particles serving as separators. This combination enhanced the availability of active binding sites, promoting more effective interactions (Fig. 2A). The method achieved a sensitivity of 0.03 ng/ml, surpassing the traditional chemiluminescence method’s sensitivity of 0.05 ng/ml. This level of sensitivity is well-suited for clinical applications. Additionally, the meticulous magnetic separation and cleaning process led to a substantial enhancement in detection specificity [38]. Another study showcased a magnetic bead-based electrochemical immunoassay for PCT determination in human serum samples, employing disposable screen-printed carbon electrode (SPE-C) and electro-kinetically driven microfluidic chips with integrated Au electrodes (EMC-Au) methods with varying detection limits (Fig. 2B) [39]. The limit of detection (LOD) is 0.1 ng/ml for SPE-C and 0.04 ng/ml for EMC-Au. Hence, the cutoff value as 0.5 ng/ml was applicable to clinically relevant concentrations of SPE-C and EMC-Au at 0.5 to 1,000 and 0.1 to 20 ng/ml, respectively. An electrochemical immunosensor (EIS) utilizing a CeO_2_CuO-Au platform, consisting of gold nanoparticle (AuNP)-modified CeO_2_-CuO, demonstrates large surface area, good biocompatibility, and high activity, enabling enhanced molecule immobilization. Thionine-modified Au@Ag heterojunction nanorods (Au@Ag-Th) were used as signal tags, which provided good active sites for connecting secondary antibodies (Fig. 2C) [40]. Additionally, a bioactive protective electro-chemiluminescent biosensor using gold nanoclusters as luminescent agent and Cu_2_S snowflakes as synergistic reaction accelerator has also been studied for PCT analysis. Cu_2_S, known for superior conductivity and a high specific surface area, served as an ideal substrate for immobilizing antibodies and as a promoter for synergistic reactions (Fig. 2D) [41].

**Fig. 3.**
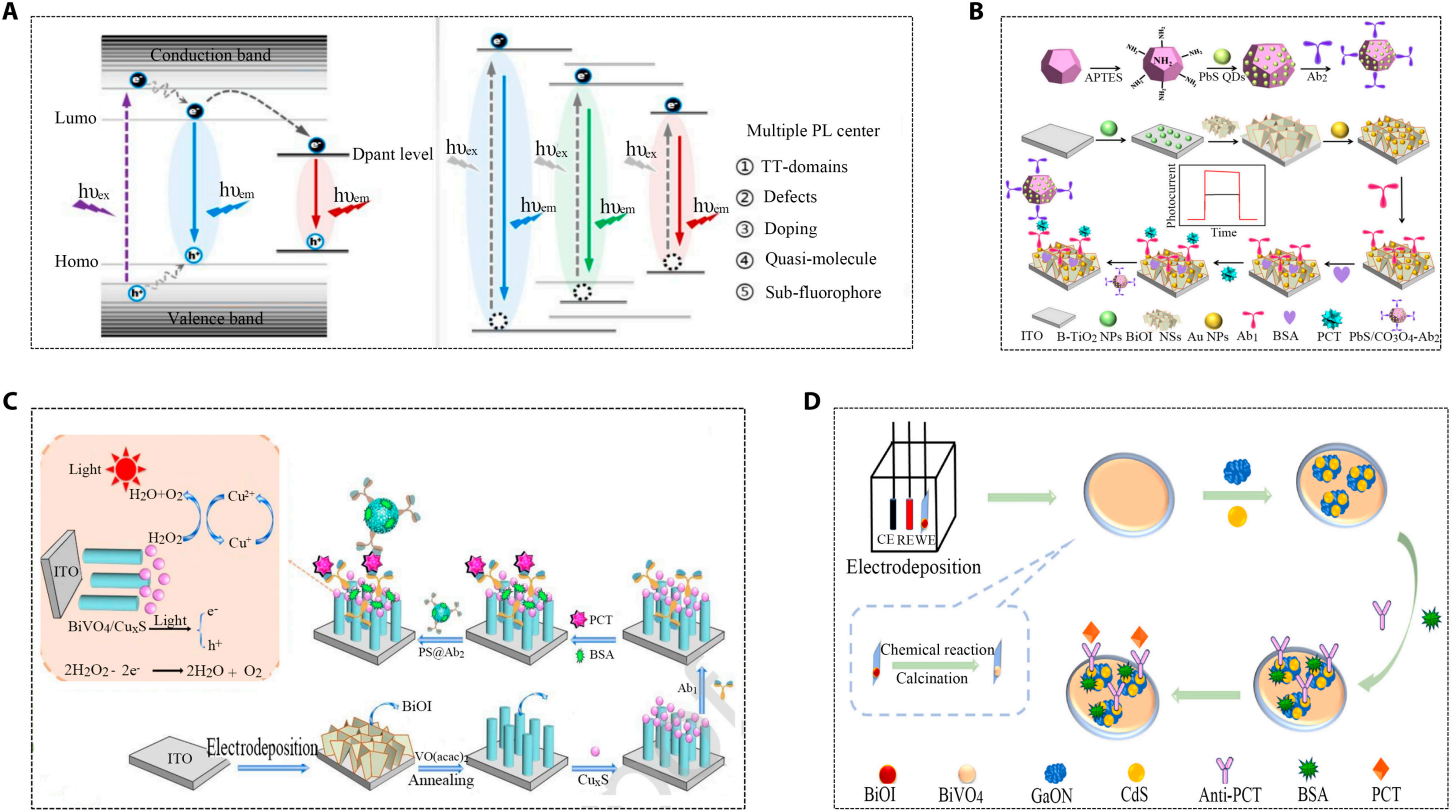
Semiconductor nanocrystal chemiluminescence sensors for PCT detection. (A) Comparison of the luminescence properties of quantum dots and carbon-based nanodots [44]. Copyright 2019 American Chemical Society. (B) Synthesis of iron PbS/Co_3_O_4_-Ab_2_ and PCT signal-off photoelectrochemical (PEC) immunosensors [45]. Copyright 2019 Elsevier. (C) Schematic of proposed electrochemical catalysis-assisted self-enhancing-based photoelectrochemical (ECASE-based PEC) sandwich-type immunosensor [46]. Copyright 2020 Elsevier. (D) Schematic illustration of PEC immunosensor [47]. Copyright 2021 Elsevier.

Semiconductor nanocrystals offer potential benefits as chemiluminescent emitters, showing ease of multicolor labeling in the visible and near-infrared spectral regions [42,43]. Despite potential toxicity and higher costs, introducing nanomaterials (MNPs, QDs, and carbon nanomaterials) into CL was a crucial strategy for signal amplification and developing various analytical methods. Studies have compared the luminescence properties of common QDs (Fig. 3A) [44]. PbS QDs were utilized as a p-type narrow bandgap semiconductor, while PbS/Co_3_O_4_ was used as a signal marker to detect PCT, and signal amplification was achieved through the collective effect of spatial impedance competing with light and electron donors (Fig. 3B) [45]. BiVO_4_ is an n-type semiconductor with superior performance and poses inherent characteristics such as strong visible light absorption and medium band gap. As an emerging biosensor material, the multi-void nanoarray BiVO_4_/Cu_x_S system was reported as signal amplification for ultrasensitive photo-immunoassay of PCT (Fig. 3C). Due to the well-matched energy levels between BiVO_4_ and Cu_x_S, porous nanoarray BiVO_4_/Cu_x_S electrodes can enhance the separation of e^−^/h^+^ pairs under visible light irradiation. In addition, BiVO_4_ porous nanoarrays had a larger specific surface area and more reaction sites, which accelerated the electron transfer rate and thus enhanced the chemiluminescence reaction [46]. Another work combined BiVO_4_ with 2 narrow bandgap semiconductors to prepare BiVO_4_/GaON/CdS ternary nanocomposites, leading to improved light absorption capabilities and higher photoelectric conversion efficiency (Fig. 3D). The addition of GaON greatly improved the charge transfer efficiency, and the in situ growth of CdS on BiVO_4_/GaON composites expanded the light absorption range and further enhanced the chemiluminescence reaction [47]. The toxicity of semiconductors was often associated with their small size, high specific surface area, and unique chemical properties. When these nanocrystals entered the organism, they might interact with biomolecules inside the cell, causing cell damage or dysfunction, but this disadvantage is not serious when tested *in vitro*. By developing straightforward, cost-effective synthesis methods and establishing optimized production processes and parameters, we aim to achieve large-scale manufacturing of semiconductor nanocrystals, thereby reducing production costs and facilitating their widespread application.

**Fig. 4.**
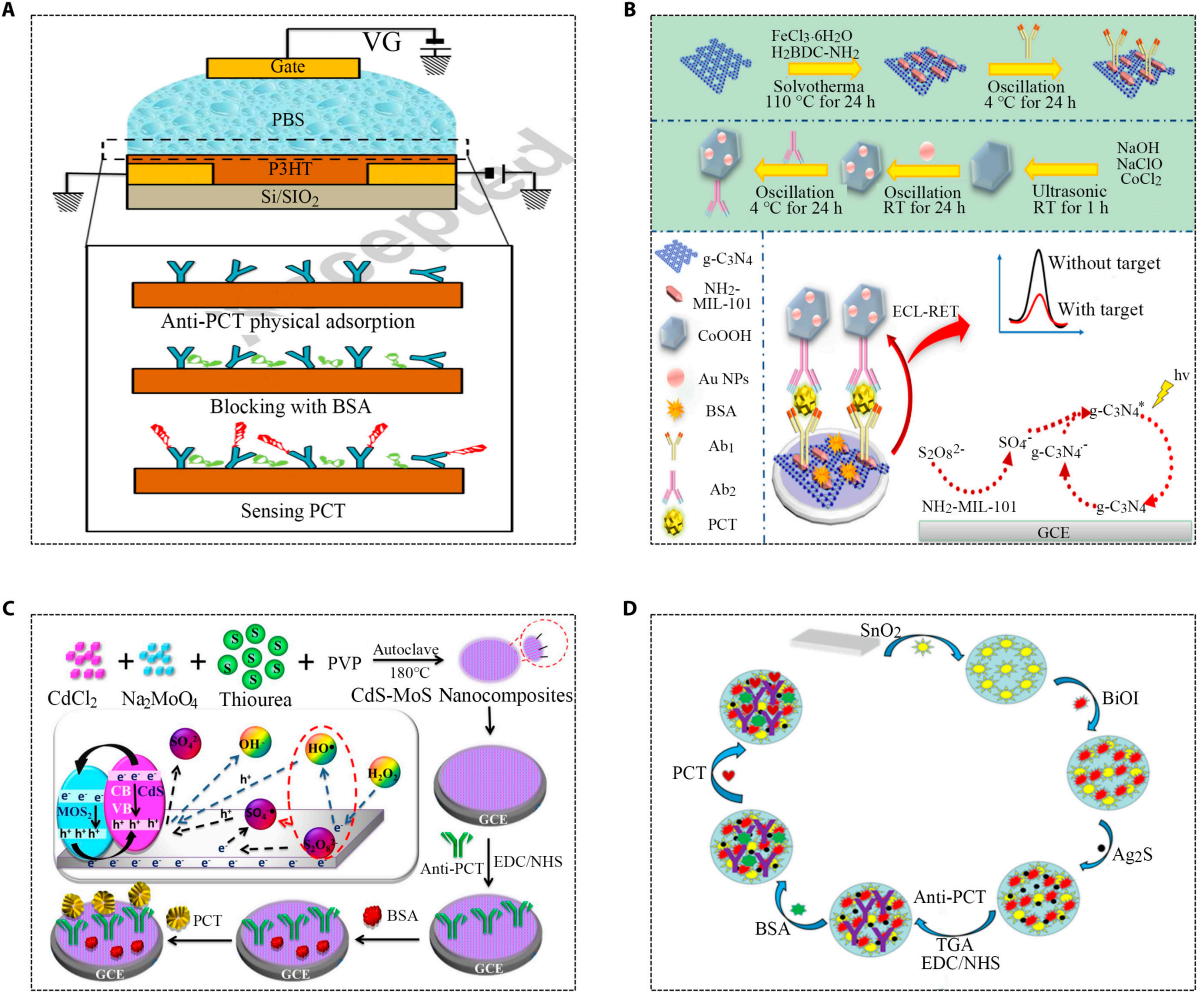
Applications of semiconductor composite materials in chemiluminescence for PCT detection. (A) EGOFET immunosensor system for PCT detection [48]. Copyright 2018 Elsevier. (B) Illustration of electrochemiluminescence immunosensor detection principle [49]. Copyright 2021 Elsevier. (C) Schematic diagram of the synergistic mechanism of double co-reactants and immunoassay [50]. Copyright 2018 IOP Publishing. (D) Illustration of the detection flow of PCT immunosensor [51]. Copyright 2021 MDPI.

Several studies have employed electronic sensors based on electrolyte-gated organic field-effect transistor (EGOFET) to detect PCT. Specific antibodies were fixed on the surface of poly3-hexylthiophene (P3HT) organic semiconductors in this immunosensor, achieving a remarkably low LOD of 2.2 pM (Fig. 4A) [48]. Chromophore graphitic carbon nitride (g-C_3_N_4_) is a metal-free semiconductor nanomaterial known for its stable properties, unique energy band structure, biocompatibility, eco-friendliness, and ease of functionalization. Loading g-C_3_N_4_ onto metal-organic frameworks (MOF) materials enhances the ECL signal’s strength and stability. The double quenching effect of the luminophore was also utilized to give it specific selectivity (Fig. 4B) [49]. The combination of CdS and other semiconductors could efficiently enhance ECL performance and photocatalytic activity. An ECL immunosensor based on CdS-MoS_2_ nanocomposite showed high sensitivity, which used H_2_O_2_ and K_2_S_2_O_8_ as dual reactants, producing more intense ECL emissions than K_2_S_2_O_8_ or H_2_O_2_ as separate reactants (Fig. 4C) [50]. Tin oxide (SnO_2_) has become a representative semiconductor material due to its unique catalytic and electrochemical properties. A new label-free photoelectrochemical (PEC) immunosensor utilizing SnO_2_ achieved highly sensitive PCT analysis, which employed SnO_2_/BiOI/Ag_2_S composite material with superior photoelectric activity as the substrate. The energy levels of SnO_2_, BiOI, and Ag_2_S matched and posed excellent light absorption properties, thus providing a strong and stable signal (Fig. 3D) [51].

Although various nanomaterials primarily function as individual catalysts in chemiluminescence reactions, the mechanisms underlying chemiluminescence enhancement remain unclear and sometimes contradictory. Investigating how nanomaterials of different sizes impact the intensity, wavelength, and kinetics of chemiluminescence represents a crucial avenue for future research in this field. Advanced characterization techniques and time-resolved spectroscopy allow direct observation of the electron transfer processes involving nanomaterials during chemiluminescence reactions. Additionally, quantum chemical calculations and simulations provide an in-depth understanding of electron transfer dynamics and electronic state changes occurring at the surface of nanomaterials. Researchers have also explored the influence of surface properties on chemiluminescence reactions. Developing more accurate theoretical models and computational methods will enable us to predict and explain nanomaterial behavior in chemiluminescence reactions. The enhancement mechanisms of nanomaterials in chemiluminescence reactions constitute an interdisciplinary research field that necessitates collaboration across disciplines such as chemistry, physics, materials science, biology, and computational science. By delving deeply into these areas, we anticipate generating novel ideas and methodologies for applying nanomaterials in the realm of chemiluminescence.

**Fig. 5.**
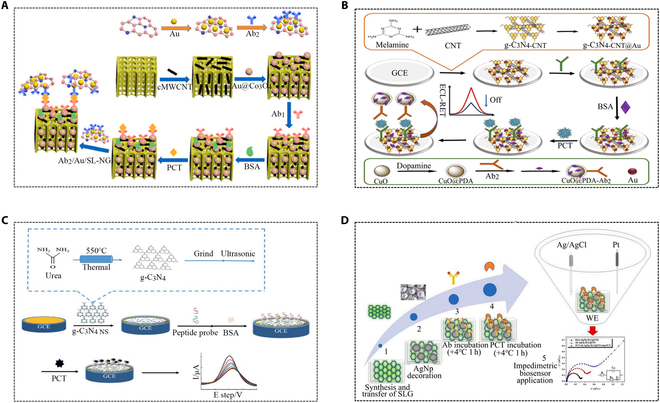
Applications of carbon materials and graphene in chemiluminescence for PCT detection. (A) Preparation procedure of 3D carbonized wood-based integrated immunosensor [52]. Copyright 2022 Elsevier. (B) Preparation procedure of the g-C_3_N_4_-CNT@Au and CuO@PDA-Ab_2_ conjugate and manufacturing process of PCT immunosensors [54]. Copyright 2020 American Chemical Society. (C) Schematic diagram of preparation of PCT electrochemical biosensor [56]. Copyright 2022 Royal Society of Chemistry. (D) Impedimetric biosensor application diagram [55]. Copyright 2023 Elsevier.

Carbon materials, with advantages like good biocompatibility and low toxicity, were utilized in an innovative 3-dimensional (3D) carbonized wood integrated EIS for ultrasensitive PCT testing. The novel 3D carbon immunosensor consisted of 3D carbonized wood, carboxyl multi-wall carbon nanotubes (CNTs), Au@Co_3_O_4_ core-shell nanospheres, and Au single-layer nitrogen-doped graphene. It had abundant interlaced microchannels, which promoted the transfer of reactants and electrons, greatly amplified the current intensity, and enhanced the stability of the electrode (Fig. 5A) [52]. Secondary antibody (Ab_2_) was immobilized using carbon quantum dots (CQDs) and AuNP coupled polyethyleneimine functionalized graphene oxide (PEI-GO) complexes. Under the combined effect of silver nanoparticles (AgNPs), polydopamine, AuNPs, and PEI-GO, the ECL signal of CQDs was significantly improved. This conductive material enhances electron transmission efficiency, further improving electrochemical sensing performance [53]. There is also a study using graphitic carbon nitride (g-C_3_N_4_-CNT@Au) functionalized with CNTs and AuNPs as the donor of ECL. The g-C_3_N_4_-CNT@Au showed better performance than pure g-C_3_N_4_ and a higher and more stable ECL signal. CuO nanospheres (CuO@PDA) covered with a polydopamine (PDA) layer were used as ECL receptors. A sandwich electrochemiluminescence (ECL) immunosensor based on ECL resonance energy transfer (ECL-ret) was developed for detecting PCT (Fig. 5B) [54]. A AgNp-doped graphene impedance biosensor achieved sensitive detection of PCT. This study utilized cost-effective and environmentally friendly materials, yielding a reusable curve with sensitivity as low as 0.55 ng/ml. The sensitivity was due to the uniform and huge surface area of single-layer graphene (SLG) and AgNp (Fig. 5D) [55]. Additionally, a label-free ultrasensitive electrochemical sensor based on graphite nitride nanosheets (g-C_3_N_4_ NS) was used to detect PCT. The probe peptide (PP) is connected to the electrode surface through π-π stacking between g-C_3_N_4_ NS and phenylalanine at the end of the peptide, providing efficient electrochemical solutions for ultrasensitive detection of PCT (Fig. 5C) [56]. An ultrasensitive sandwich electrochemical method was employed for PCT testing. First, a reduced graphene oxide (rGO)–gold (Au) nanocomposite film was assembled as an immunosensor to improve the immobilization of PCT antibody 1 (Ab1). Next, single-walled carbon nanohorns/hollow platinum chains complexes labeled with PCT Ab2 served as signal tags [57]. Another study enabled simultaneous detection by combining a nanocomposite surface composed of cross-linked bovine serum albumin with a reduced graphene oxide nanoparticle structure that not only protects against biological contaminants but also maintains conductivity for multiple sepsis biomarkers [58].

**Fig. 6.**
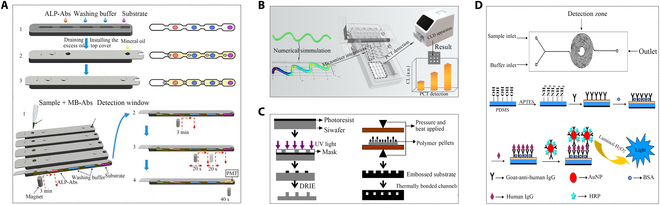
Applications of the microfluidics technique in chemiluminescence for PCT detection. (A) Diagram illustrating operation of the microfluidic chip and workflow of the microfluidic CLIA [61]. Copyright 2022 Elsevier. (B) Design of DHMC with excellent mixing effects and their use in PCT determination [60]. Copyright 2023 Royal Society of Chemistry. (C) Fabrication process steps for plastic microfluidic chip [62]. Copyright 2007 Springer Nature. (D) Schematic diagram of PDMS microfluidic device and immunoassay procedure [63]. Copyright 2016 Elsevier.

Compared to single-analyte immunoassays, multiplexed analysis emerges as a promising trend in sample analysis. Recently, CLIA has been employed for multiplexed analysis on microfluidic chips. A newly developed PCT diagnostic kit utilized a one-step sandwich CLIA approach for quantitative detection of serum concentration of PCT using the HYBIOME e-180 system. Achieving a low LOD of 0.0075 ng/ml, the within- and between-kit coefficient of variation ranged from 0.8% to 3.9%, and the detection range is 0.01 to 110 ng/ml [59]. An ideal hyperbolic microfluidic chip (DHMC) was employed for the sensitive detection of the inflammatory marker PCT via chemiluminescence signal. It is simply achieved through mixing of specimens to be tested and detection reagents (Fig. 6B); this approach offered enhanced precision [60]. Another innovative method involved a CLIA based on active droplet array microfluidics, enabling rapid PCT detection within 12 min (Fig. 6A) [61]. Using a polyolefin microfluidic chip prepared through a silicon master mold hot pressing method, disease biomarkers, including C-reactive protein (CRP) in the same class as PCT, were detected in human serum (Fig. 6C) [62]. Additionally, a polydimethylsiloxane (PDMS) microfluidic chemiluminescence device with signal amplification capability, featuring a double-helical flow channel, was developed for specific detection of human immunoglobulin G (IgG), and it was generalizable in PCT testing (Fig. 6D) [63]. The future of microfluidic chips hinges on portability, integration, and automation development, facilitating the automation of CLIAs.

## IF for PCT Detection

IF, an early development in labeling immune technology, is a recognized quantitative immune detection method. The fluorescent substances that can be used as markers should produce obvious fluorescence and do not affect the binding activity of antigens and antibodies. The commonly seen options include fluorescein isothiocyanate, tetraethyl rhodamine, tetramethyl rhodamine isothiocyanate, 4-methylumbelliferone-β-D galactopyranoside, and lanthanide metals. It can be divided into direct fluorescence (DIF) and indirect fluorescence (IIF) subtypes. DIF makes use of fluorescent labeled antibodies to directly bind to the target antigen [64]. IIF often adopts a 2-step route, in which an unlabeled primary antibody binds to the antibody target followed by detecting it via a fluorophore labeled second antibody. The extra binding step promises high sensitivity due to the further amplification of the fluorescence signal, compensating for its complexity and being time-consuming [65,66]. Quantitative immunofluorescence assay (IFA) is cost-effective, rapid, and user-friendly, and offers improved diagnostic capabilities for detecting PCT. Its detection range is 0.1 to 100.0 ng/ml [67].

**Fig. 7.**
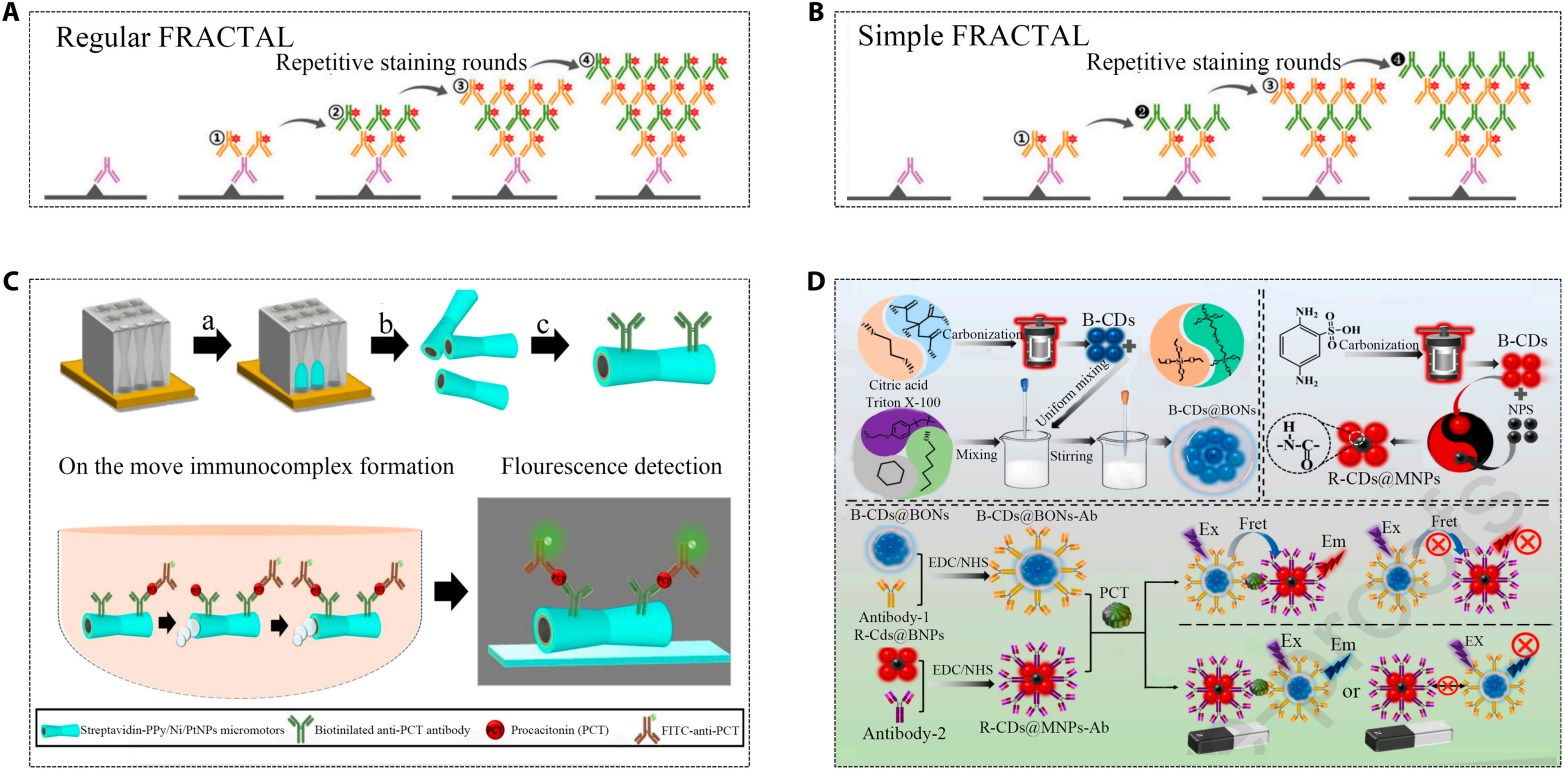
Representative demonstrations of novel immunofluorescence sensors for PCT detection. (A and B) Mechanism schematics of regular and simple fractal [69]. Copyright 2009 Royal Society of Chemistry. (C) Preparation system of anti-PCT-PPy/Ni/PtNPs micromotors (PPy: polymeric polypyrrole outer layer; PtNPs: Pt nanoparticles) [68]. Copyright 2022 American Chemical Society. (D) Preparation of immunofluorescent nanocapsules and immunomagnetic carbon dots and 2 sensing systems for PCT detection [70]. Copyright 2023 Elsevier.

To meet the growing demand for point-of-care (POC) PCT detection in clinical applications, a micromotor-based fluorescence immunoassay was developed for PCT testing. The lowest detection limit achieved was 0.07 ng/ml, with a detection concentration range of 0.5–150 ng/ml, and requiring only 25 μl of sample (Fig. 7C); this technology addressed the need for high sensitivity [68]. Signal intensity in immunofluorescence detection could be enhanced by over 9 times through circulating staining with secondary antibodies (Fig. 7A and B) [69]. A fluorescence amplified PCT biosensing system uses nanocapsules and magnetic carbon dots. Nanocapsules ensured the effective encapsulation of nanoparticles and prevented leakage, and also greatly improved the enrichment ability of fluorescent nanoparticles to amplify the fluorescence signal. This strategy enables trace-level quantification of PCT within a detection range of 1 to 1,000 pg/ml (Fig. 7D) [70]. In PCT detection, paired mouse mAbs were usually used to bind to 2 different antigen binding sites, thereby forming a sandwich complex. When fixing the fluorescent labeled antibody on the testing tube wall, the content of the PCT marker reflected by a luminous reagent can be obtained quantitatively with comparison to the standard curves. Like chemiluminescence, immunofluorescence emits light due to the transition of molecular energy level back to the ground state; meanwhile, the fluorescence needs to provide an external light source, that is, photoluminescence. Ultraviolet light is usually used as an external light source. Upon absorbing it, the substance emits fluorescence in the visible band.

However, compared with the common fluorophore whose emission wavelength is in the ultraviolet (UV) region, the fluorescein secondary antibody in the near-infrared region (NIR) has a higher signal-to-noise ratio and other important advantages. NIR fluorophore with high chemical stability, light stability, strong target specificity, and high fluorescence brightness has been developed, which is expected to enter clinical applications in the future [71]. A recent work introduces a AuNP-assisted immunofluorescence method, utilizing a second antibody functionalized dye in a sandwich structure assembly with antibody-functionalized AuNPs and antigens. It can realize specific and sensitive detection of biomarkers without the phenomenon of front and back banding. In addition, the method does not require sample handling and washing steps, which greatly saves detection time. This homogeneous multicomponent fluorescence method significantly improves sensitivity and dynamic range [72], though its application to PCT detection has not yet been explored.

## ELISA for PCT Detection

**Fig. 8.**
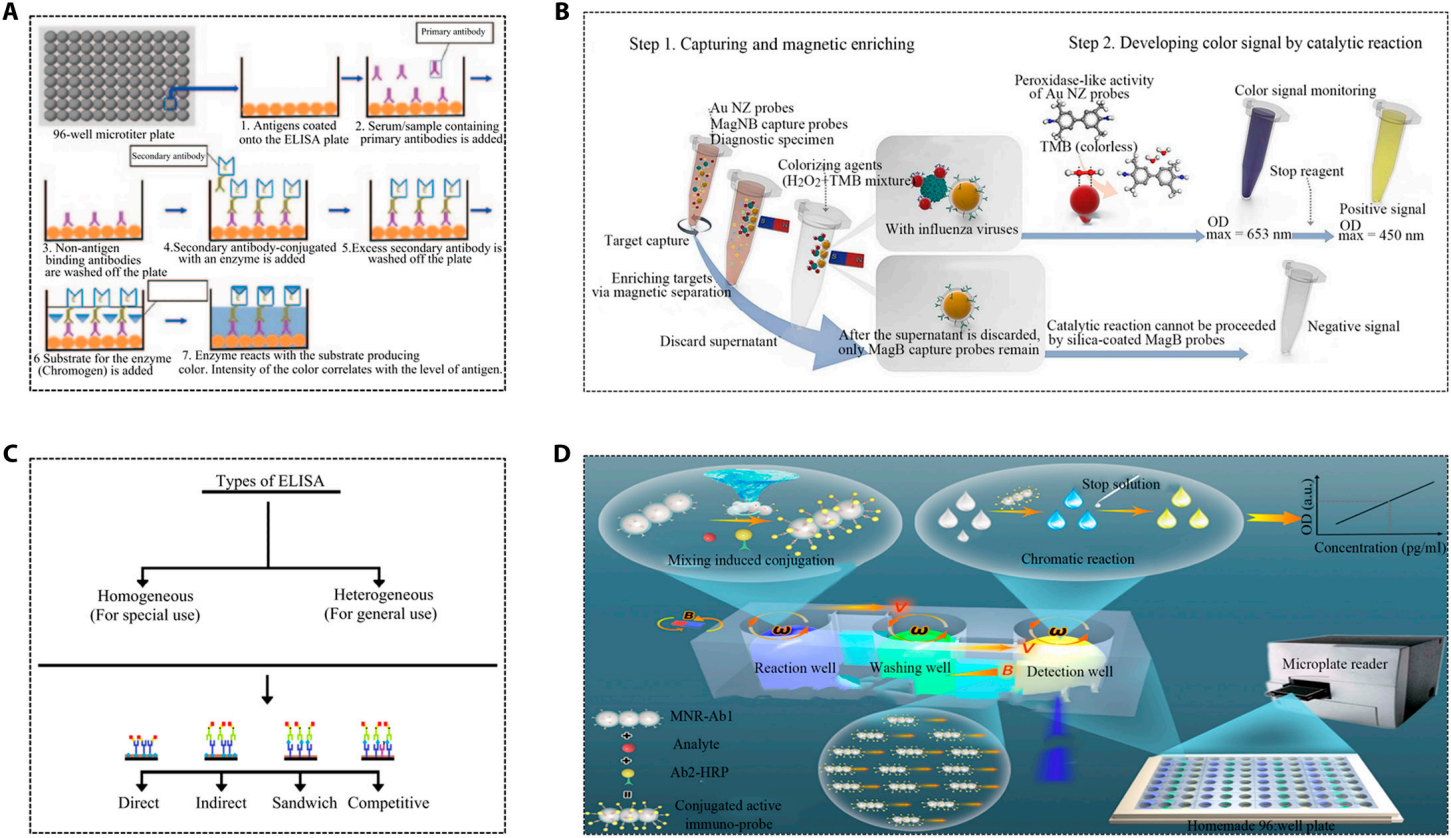
Applications of magnetic materials usage in ELISA for PCT detection. (A) ELISA technique used to detect an antigen in a given sample [73]. Copyright 2013 Elsevier. (B) Working principle of quantitative detection of colorimetric diagnostic kit based on MagLISA [81]. Copyright 2018 American Chemical Society. (C) Different ELISA test types [75]. Copyright 2015 Elsevier. (D) Magnetic nanorobot-enabled automated and efficient ELISA [83]. Copyright 2022 American Chemical Society.

In 1971, ELISA was initially proposed for quantifying IgG-type immunoglobulins. ELISA, a direct or indirect method for detecting antigens or antibodies, involves attaching them to the surface of micropores (Fig. 8A) [73,74]. Different detection methods, including direct ELISA (antigen screening), indirect ELISA, sandwich ELISA (antibody screening), and competitive ELISA (antigen/antibody screening) (Fig. 8C), were employed based on detection conditions and specific sample requirements [75]. Typically, the specific antibody was coated on the enzyme-labeled substrate and the antigen was added to the coated antibody and bound. After washing away unbound substance, another specific antibody labeled with enzyme was introduced for detection. The presence of analytes can be determined and quantified by the color changes from enzyme-linked conjugates and enzyme substrates. The specific antibody–antigen interaction promised high sensitivity at picomolar to nanomolar ranges (10^−12^ to 10^−9^ mol/L). Besides PCT, the majority of known inflammatory cytokines are also detectable and quantified with ELISA. For example, the cytokine spectrum of sepsis-infected persons tracked by ELISA helps facilitate understanding of the inflammatory changes [76]. Considering the complexity of ELISA operation steps often bringing in diverse interfering factors, a growing number of studies are aimed at simplifying these steps to reduce the influence of interfering and crosstalk issues.

The application of a streptavidin system in ELISA plays an important role in signal amplification, which has been proven to increase the number of lapels per sandwich by 3-fold [77–79]. Traditional ELISAs require long incubation times and laborious multi-step washing processes, making them inefficient and laborious. In the realm of nanotechnology in biomedicine, nanoparticles act as carriers to assist immunoassays, greatly enhancing sensitivity, simplicity, and specificity of detection [80]. For example, magnetic nanoenzyme-linked immunosorbent assay (MagLISA) combined silicon-shell MNPs and AuNPs to achieve analyte separation and enzymatic activity amplification of gold nanoenzymes (AuNZs) with ultrasensitivity (Fig. 8B) [81]. Since AuNPs have a high surface area, they can be easily coupled with biomolecules to prepare dual-labeled AuNP probes, and a detection antibody coupled with multiple HRPs on AuNPs greatly improved the detection signal. It can be directly used for PCT detection in human serum with a sensitivity of 20 pg/ml [7]. A reported sandwich ELISA for PCT quantification in equine plasma samples operated within a range of 25 to 1,000 ng/ml [82]. A rod-shaped magnetically driven nanorobot served as an operable immunoassay probe, facilitating automated and efficient ELISA analysis strategies. In gradient magnetic fields and rotating magnetic fields, directional uniform mixing was achieved, shortening incubation time and improving detection efficiency (Fig. 8D) [83].

To achieve a low LOD, a proposed fluorescence detection system employed alkaline phosphatase (ALP) as the marker enzyme and copper nanoclusters (CuNCs) as the tracer agent. This system, utilizing human IgG as the standard antigen, established a reliable detection range of 0.05 to 12 ng/ml, a much lower detection limit compared to previously reported ELISA methods [84]. Fluorescence-based oligonucleotide-linked immunosorbent assays and fluorescence-based oligonucleotide-linked immunospots provided highly multiplexed methods with signal amplification using DNA-barcoded antibodies. Signal enhancement and multi-objective simultaneous detection were successfully carried out through DNA complementary pairing and modular orthogonal DNA tandems [85]. Further data analysis indicated that ELISA outperformed IIF in detecting autoantibodies [86].

**Fig. 9.**
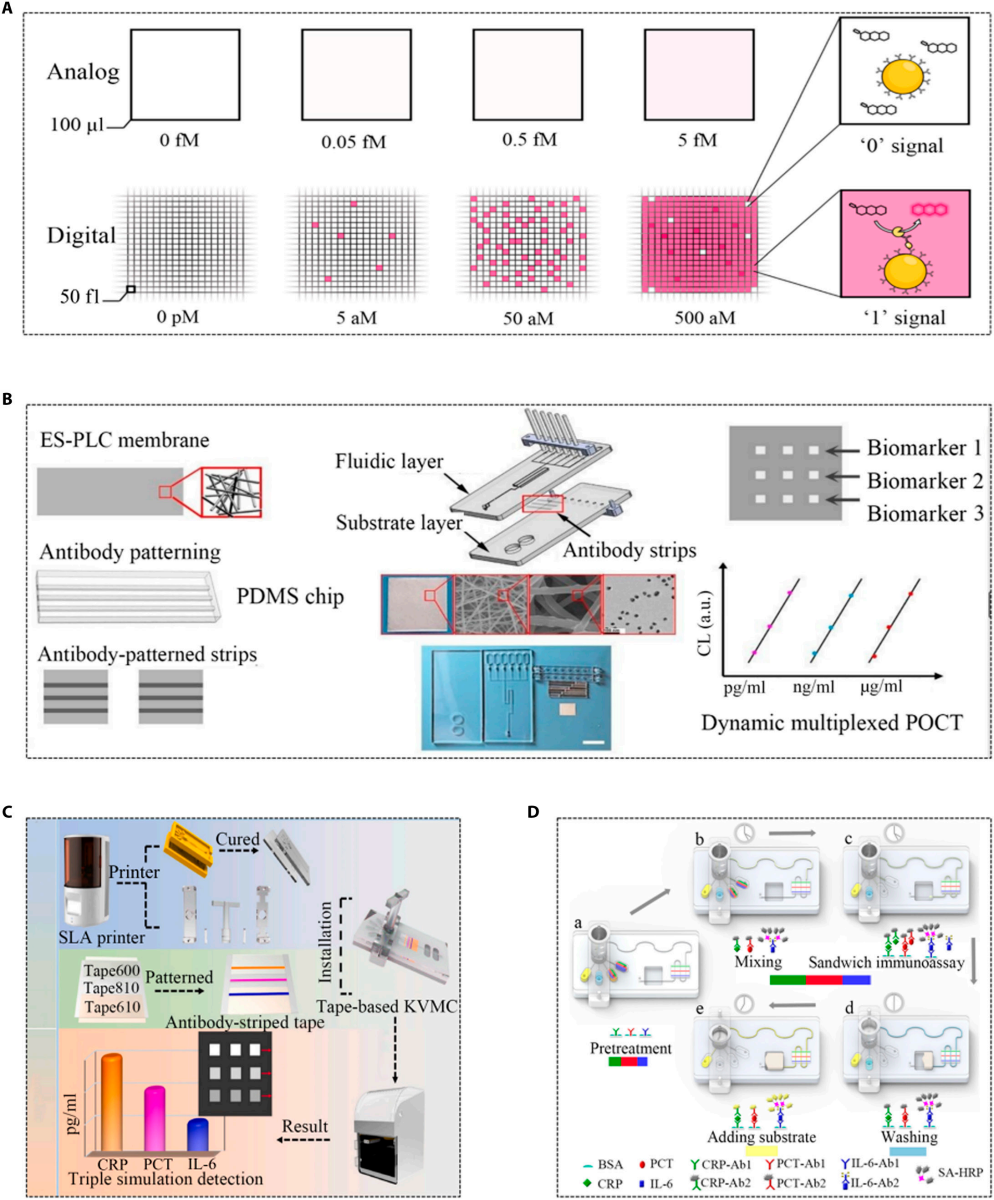
Applications of ELISA to detect PCT with the assistance of microfluidic technology. (A) Comparison of conventional ELISA and digital ELISA [87]. Copyright 2023 Royal Society of Chemistry. (B) Schematic diagram of hierarchical structure microchip detection system [89]. Copyright 2019 Royal Society of Chemistry. (C) Control tape KVMC system based on 3D printing for multi-pathway immunoassay of CRP, PCT, and interleukin-6 (IL-6) [90]. Copyright 2022 Elsevier. (D) Detection system for synchronous detection of CRP, PCT, and IL-6 using WMC-MDP [91]. Copyright 2022 BioMed Central Ltd.

Monitoring minute changes in PCT and other protein marker concentrations are of great value in clinical diagnosis and pathological research. The increasing demand for ultrasensitive and multiplexed protein detection has increased the development speed of digital immunoassay technology (Fig. 9A). Specifically, traditional (analog) ELISA cannot accurately quantify, whereas digital ELISA can calculate the average number of enzyme molecules per bead at a given sample concentration, with the red grid representing the lowest detectable concentration of the target protein in both analog and numerical methods [87]. Microfluidic systems form the basis of digital immune analysis, with relevant research already underway [21]. For instance, a microfluidic ELISA chip-based POC assay detected protein markers with a detection limit as low as 0.0005 μg/ml, demonstrating good reproducibility in intra-test (5.1%) and between-test (9.3%) CV values [88]. The hierarchical structure microchip POC immunoassay offered an adjustable detection range and could realize multiplex detection of biomarkers (Fig. 9B) [89]. A study introduced a tape-based key valve microfluidic chip (KVMC) system capable of simultaneously detecting multiple biomarkers other than PCT. A single button valve was employed to adjust the mixing and sequence of multiple reagents, resulting in higher detection efficiency and integration. The entire test was conducted in a portable automated device, meeting the need for POC testing (POCT) of infections (Fig. 9C) [90]. A waveform microfluidic chip (WMC)-assisted multiplex detection system was utilized, involving premixing of antigens and detection antibodies in a micromixer. This approach simplified incubation and washing steps, ultimately reducing the overall detection time and enhancing reproducibility through optimization and preprocessing (Fig. 9D) [91]. Microfluidic technology significantly improved the diagnostic accuracy of ELISA, reducing the disease burdens and playing a crucial role in the field of precision medicine.

## Immune Colloidal Gold Technique for PCT Detection

**Fig. 10.**
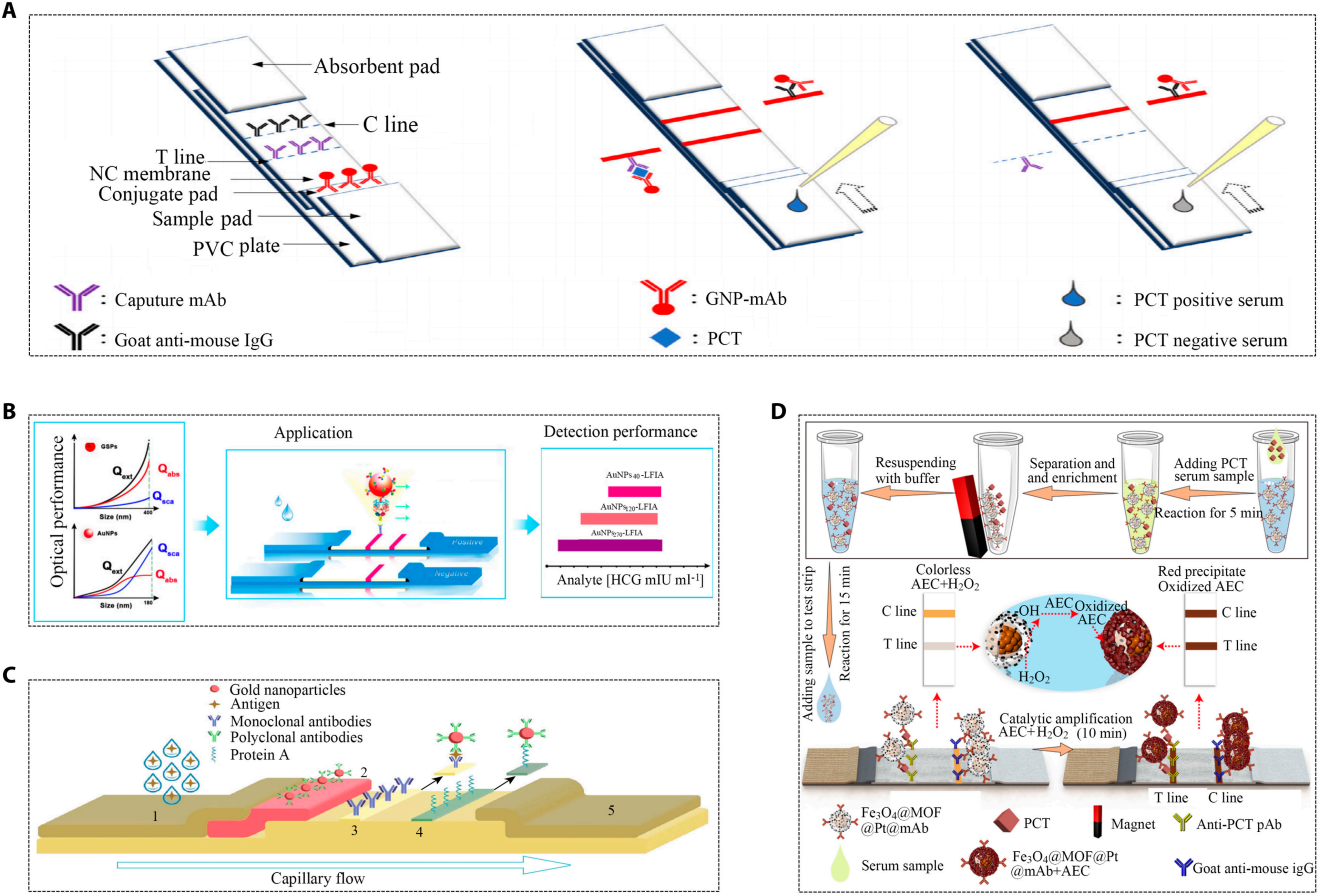
Applications of new metal nanomaterials in immunochromatography for PCT detection. (A) Paper sensor for PCT testing [97]. Copyright 2022 Elsevier. (B) Schematic illustration of GSPs-LFIA and AuNPs-LFIA [93]. Copyright 2020 PubMed Central. (C) Scheme of LFIA using gold nanoparticles as labels [100]. Copyright 2018 Springer Nature. (D) Schematic illustration of Fe_3_O_4_@MOF@Pt-immunolabeled lateral flow immunoassay [99]. Copyright 2022 American Chemical Society.

AuNPs are renowned for their superior chemical stability and size-dependent optical properties [92]. Immune colloidal gold was developed as a lateral flow immunochromatography assay (LFIA) determination technology, integrating the double antibody sandwich principle [93–95]. In clinically applied immunochromatography, the lowest reported detection limit for PCT is approximately 0.1 ng/ml. Despite providing fast (usually less than half an hour) and simple PCT detection, achieving quantitative results remains a challenge [96]. A gold-based paper sensor designed to detect PCT in clinical samples demonstrated a detection limit of 0.1 ng/ml and a detection range from 0.49 to 13.90 ng/ml (Fig. 10A) [97]. To enhance sensitivity, researchers explored increasing the size of AuNPs. Studies indicated that larger-sized AuNPs have higher optical intensity, but excessive size diminished sensitivity due to reduced diffusivity and stronger light scattering. Small-size (12 nm) hydrophobic AuNPs were found to self-assemble into large-size (100 to 400 nm) gold superparticles (GSPs), demonstrating strong light absorption and extremely weak light scattering, achieving quantitative detection with improved sensitivity (Fig. 10B) [93].

A study has developed a composite nanomaterial based on colloidal gold–colloidal gold enhancer (CGE). Colloidal gold and CGE served as tracers, labeling antibodies to form immunoprobes for subsequent serum PCT qualification. The absorption coefficient of CGE at 520 nm was found to be approximately 10 times that of colloidal gold immunoprobes, significantly improving the sensitivity of detecting PCT [98]. Another study confirmed the high sensitivity of CGE immunoprobe detection, with detection limits of 0.5 ng/ml for colloidal gold and 0.025 ng/ml for CGE immunoprobes. Notably, the CGE immunoprobe demonstrated a sensitivity 20 times higher than that of the colloidal gold immunoprobe [98]. Additionally, a “three-in-one” multifunctional catalytic colorimetric nanohybrid (Fe_3_O_4_@MOF@Pt) composed of Fe_3_O_4_ nanoparticles, MIL-100 (Fe), and platinum (Pt) nanoparticles overcame the signal intensity limitation of AuNPs in colorimetric assays. It exhibited high colorimetric signal brightness and ultra-high peroxide simulation activity, and achieved a rapid magnetic response approximately 2,280 times more sensitive than normal LFIA. It overcomes the shortcoming of insufficient colorimetric signal intensity of AuNPs and achieves ultrasensitive analysis of PCT on an immunochromatography platform (Fig. 10D) [99]. Besides composite materials, studies have explored different types of AuNPs, such as gold nanospheres (GNSs), gold nanopopcorns (GNPNs), and gold nanostars (GNSTs), using them as markers in immunochromatography. They both exhibited enhanced stability due to a complex 3-dimensional structure. Furthermore, their spherical shape and vast surface area facilitated the presence of surface-bound antibodies, thereby increasing the detection sensitivity. Importantly, this system requires no additional steps, thereby avoiding extra consumption of antibodies. The silver enhancement method was employed to improve sensitivity. This method’s sensitivity for detecting PCT was 10 times that of the conventional colloidal gold immunochromatographic assay labeled with gold nanospheres (Fig. 10C) [100].

**Fig. 11.**
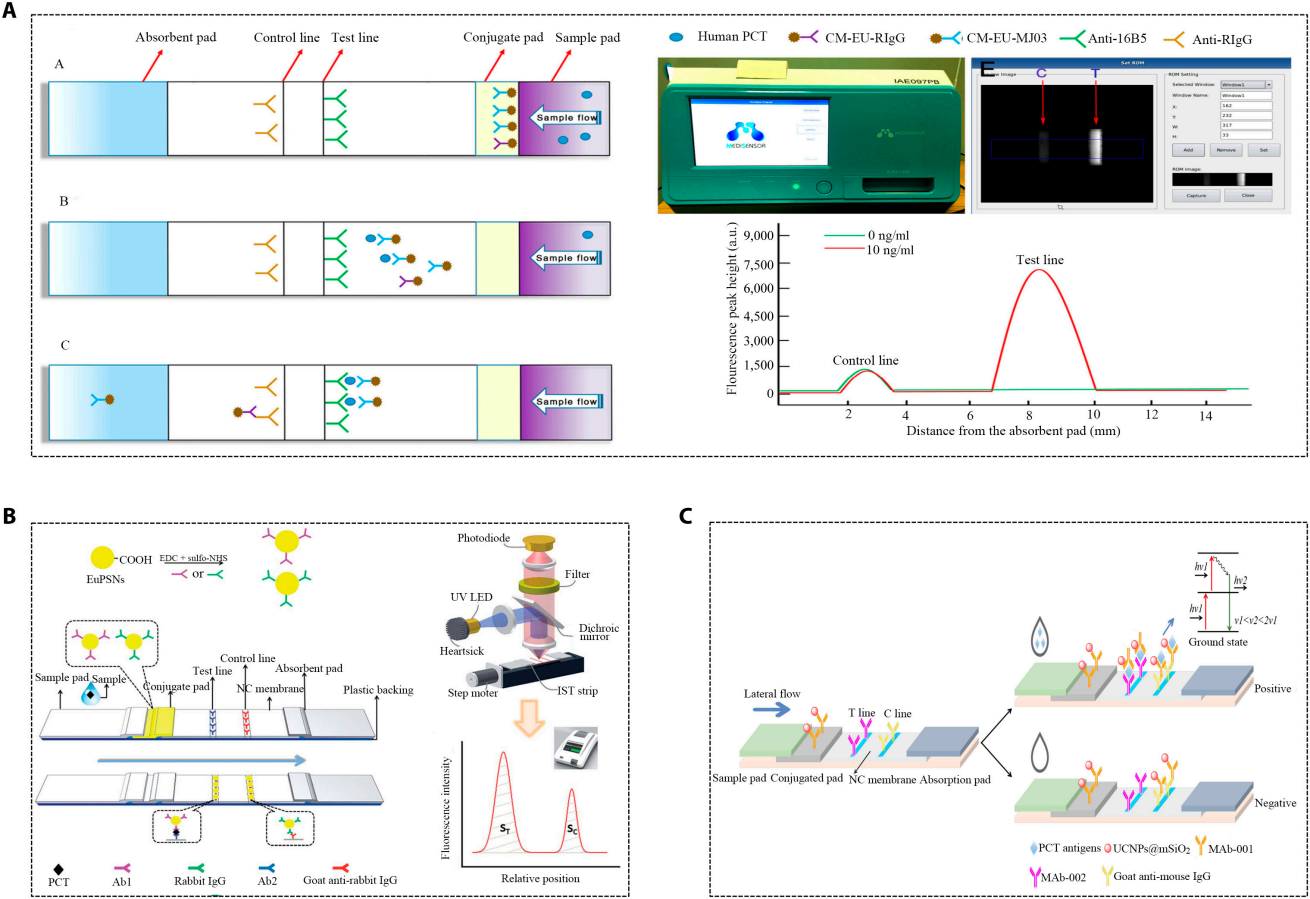
Applications of special markers in immunochromatography for PCT detection. (A) Schematic system of the assay procedure [103]. Copyright 2017 MDPI. (B) PCT quantitative analysis process by (dibenzoylmethane)mono(1,10-phenanthroline)europium(III) into monodisperse PS nanoparticles (EuPSNs)-based immunochromatographic strip test (IST) [104]. Copyright 2016 Royal Society of Chemistry. (C) Schematic illustration of sandwich format LFIA in PCT testing and upconversion luminescence energy changes [105]. Copyright 2021 IEEE.

A PCT assay utilizing a biotin–streptavidin system enhanced transverse flow immunoassay. The amplification of the analysis signal resulted from the accumulation of AuNP clumps [101]. Another platinum staining method based on a test strip retained the original properties of colloidal gold while leveraging platinum nanoparticles (PTNPs) with good catalytic activity as a signal label, achieving sensitive and quantitative testing [102]. In addition to gold nanomaterials, studies also have explored lanthanide chelates as fluorescent markers for lateral flow immunoassays. Carboxylic acid-modified europium (III) (Eu (III)) chelate particles were used to manufacture immunochromatography test strips, exhibiting a narrow emission spectrum and a wide excitation spectrum (613 nm and 333 nm). Combined with a portable time-resolved fluorescence reading system, this approach offered a broader linear range, and greater sensitivity and accuracy, enabling instant POCT detection (Fig. 11A) [103]. In another work, lanthanide chelates encapsulated polystyrene nanoparticles for rapid quantitative immunochromatographic analysis of PCT, shortening the detection time to 15 min (Fig. 11B) [104]. An immunochromatography-based upconversion luminescence (UCL) system utilized mesoporous silica-encapsulated upconversion nanoparticles (UCNPs@mSiO_2_) as probes. The biocompatibility and strong luminescence intensity of UCNPs@mSiO_2_ made it an ideal choice for the quantitative analysis of PCT in plasma (Fig. 11C) [105].

**Fig. 12.**
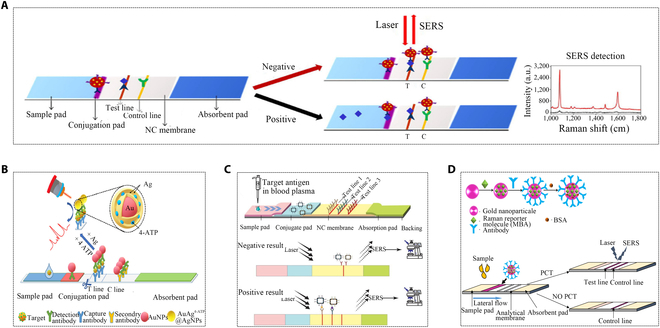
Applications of SERS in immunochromatography for PCT detection. (A) Schematic representation of LFIA bands and SERS-LFIA bands [107]. Copyright 2017 Elsevier. (B) Schematic diagram of i-SERS [109]. Copyright 2021 Springer Nature. (C) Reaction results of SERS-ICA (immunochromatography) strips and composition of the SERS-ICA system [110]. Copyright 2020 Royal Society of Chemistry. (D) Formation of Ab-Au-MBA immunoprobe and LFIA strategy based on SERS detection (MBA: 4-mercaptobenzoic acid) [111]. Copyright 2016 Trans Tech Publications Ltd.

The development of colloidal gold immune technology has overcome the limitations of traditional colloidal gold strips, addressing issues such as low sensitivity and non-quantification. SERS has extended the application of colloidal gold, demonstrating marked signal amplification when Raman signal molecules were labeled on the colloidal gold surface (Fig. 12A) [106,107]. This characteristic allows for quantitative or semi-quantitative detection of PCT. In a particular study, dual-channel detection was achieved by embedding 2 different Raman reporter molecules in the Au@Ag shell. Simultaneously, magnetic beads facilitated rapid capture [108]. However, this method has not been applied in immunochromatography, considering introducing complexities to the detection process. Due to the low stability of nanoparticles labeled with Raman reporter molecules, an in situ Raman enhancement (i-SERS) method was developed. This method, generating SERS signals on AuNPs, not only retained the previous advantages of colloidal gold strips but also achieved better sensitivity and quantitative detection results, which results in a sensitivity of 0.03 ng/ml and a turnaround time of 16 min (Fig. 12B) [109]. There is also a work that combines SERS with immunochromatography to establish a dual-pathway detection immunochromatography. IL-6 antibodies and PCT antibodies were coupled to gold nanocages respectively as SERS markers. With the addition of IL-6 and PCT, the reactants were captured on the 2 test articles. The special structure of graphene nanoclusters rich in biological binding sites and hot spots endows the system with extremely high SERS performance (Fig. 12C) [110]. Utilizing gold nanoflowers as labels in an immunochromatographic detection method based on photometry and SERS (Fig. 12D) [111]. Although the combination of SERS with immunochromatography offered obvious advantages for detecting PCT, it also led to complexities to some extent. These include challenges related to technology integration, substrate stability, multiple detection targets, instrument costs, operational intricacies, and issues with background interference and signal enhancement. However, as technology continues to progress and optimization efforts persist, it is anticipated that these challenges will be overcome. Consequently, this will further facilitate the application and development of SERS in the field of immunochromatography.

**Fig. 13.**
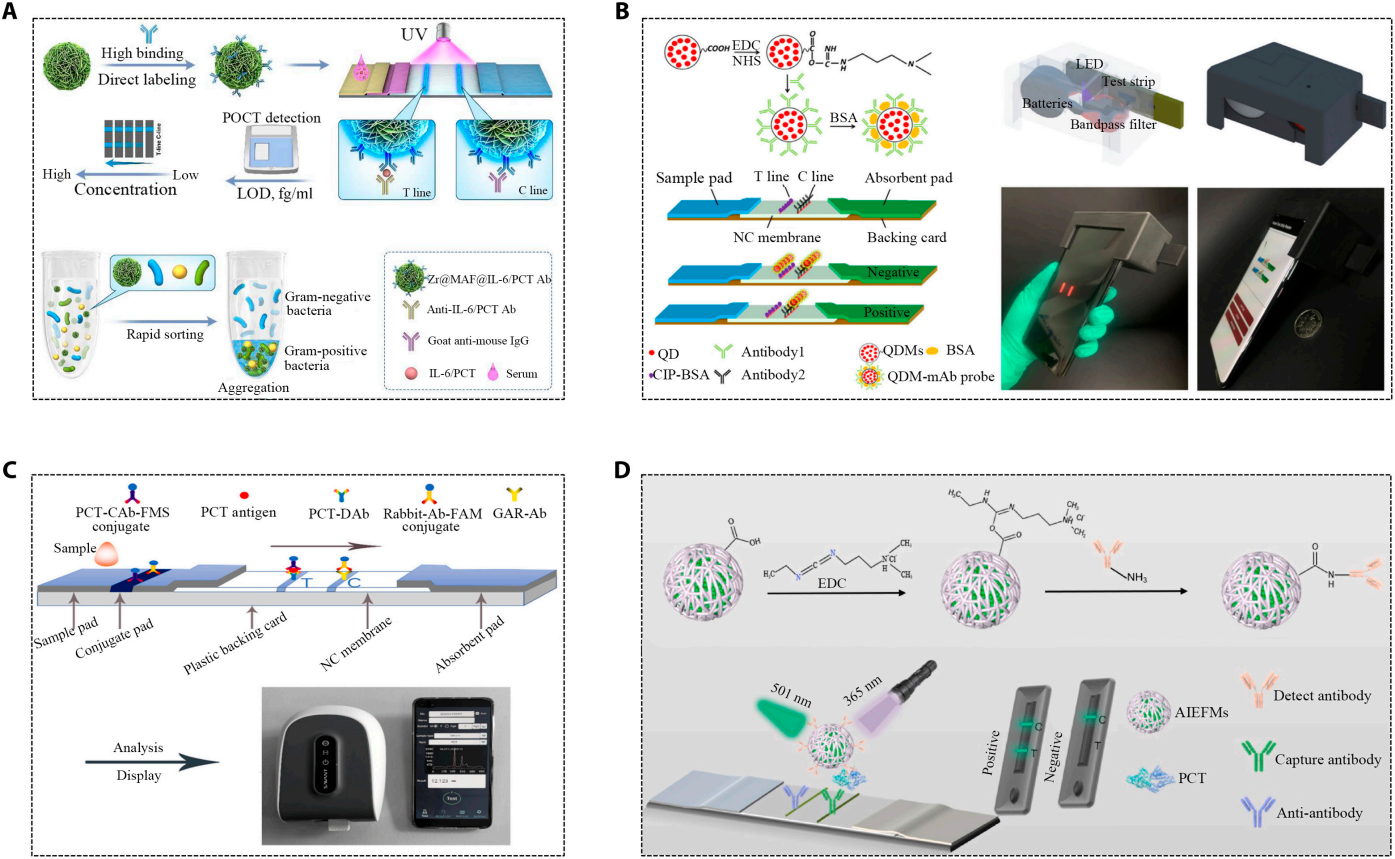
Applications of MAFs, QB, and QDM in immunochromatography for PCT detection. (A) Schematic representation of MAF-based LFA for IL-6/PCT detection and differentiation of Gram-positive bacteria from Gram-negative bacteria [112]. Copyright 2023 American Chemical Society. (B) Schematic system of the ciprofloxacin (CIP) detection using quantitative CIP test strip [113]. Copyright 2021 Elsevier. (C) Schematic diagram of test strips and handheld fluorescence immunoassay analyzer and a handheld fluorescence immunoassay analyzer controlled by mobile phone Bluetooth, used to measure the fluorescence intensity of FM complexes after lateral migration of patient serum [118]. Copyright 2021 Elsevier. (D) Schematic of the synthesis route to aggregation-induced luminescence microsphere-conjugated monoclonal antibodies (AIEFMs@mAbs) and schematic illustration of the AIEFMs-ICA sandwich format by using green-emissive AIEFMs as a signal reporter [114]. Copyright 2023 Elsevier.

Fluorescent microsphere (FM) immunochromatography has emerged as an alternative to address issues of poor sensitivity and non-quantification. A fluorescent metal-AIEgen framework (MAFs) has 2 functions: protein detection and bacterial identification. With a large surface area, excellent photostability, and multiple active sites for protein binding, MAF enabled ultrasensitive detection of multiple biomarkers in lateral flow immunoassays. Its detection sensitivity for PCT is 0.333 pg/ml, offering promising application prospects in POCT for bacterial infections (Fig. 13A) [112]. In another approach, aggregated luminescent microspheres replaced organic fluorescein dye-embedded FMs, overcoming aggregation-induced quenching and Stokes shift defects. This approach held great potential to optimize detection performance of immunochromatography platforms, as depicted in Fig. 13B to D [113,114]. Additionally, highly luminescent quantum dot beads (QBs) were prepared as an alternative to AuNPs. The fluorescence intensity of each QB was approximately 900 times that of the QD. It can be used for quantitative detection of serum PCT. Its detection limit was approximately 10 times lower than traditional AuNPs immunochromatography methods [115]. To reduce cost, rapid immunochromatographic test strips with colloidal carbon and quantum dot microspheres (QDMs) as labels have been developed. The test strip, equipped with a smart reader and mAb probe, offers fast detection, simple operation, and cost-effectiveness [11,116,117]. There is research employing fluorescent carbon dots/SiO_2_ nanospheres for lateral flow assay (LFA). Carbon colloids and CNTs served as signal reporters, providing enhanced sensitivity, stability, and environmental friendliness. Although not yet applied to PCT detection, there is optimism about its broad application prospects [117]. Additionally, a handheld FM-based PCT detection system has been developed, enabling test strips with a wide detection range and a low LOD. This method ensured accurate detection of PCT concentration in human serum within 12 min, with long-term storage stability exceeding 12 months (Fig. 11C) [118].

Overall, MAFs exhibit excellent fluorescence properties, enabling highly sensitive detection. On the other hand, AIEgen emits light selectively under specific conditions, making it useful for detecting target molecules in complex biological samples. However, it is worth noting that MAFs may not be as stable as other FMs, especially under certain conditions. Their synthesis typically involves multi-step reactions and precise control conditions, adding complexity to the preparation process. QB stands out due to its high fluorescence brightness, particularly advantageous for detecting low-concentration analytes. Additionally, the size of QB can be controlled during synthesis to tailor its fluorescence properties. However, it is essential to recognize that while QDs are generally more stable and less affected by environmental factors than organic dyes, they can be potentially toxic and lack biocompatibility. QDM, with its larger size, facilitates separation and purification from solution. Integrating multiple QDs within a single microsphere enhances the fluorescence signal and improves detection sensitivity. Structurally, QDM tends to be more stable than standalone QDs. Nevertheless, achieving uniform fluorescence properties in QDM may involve complex processes and rigorous quality control. When choosing a fluorescence microsphere detection platform, consider specific requirements for PCT detection, including sensitivity, selectivity, stability, biocompatibility, and preparation complexity.

## Turbidimetric Inhibition Immunoassay for PCT Detection

Immunoturbidimetry, a dynamic monitoring method for detecting antigen and antibody complexes, has various applications, including immune transmission turbidimetry, immune scattering turbidimetry, and immune latex turbidimetry [119–121]. Immunoturbidimetry offered both rate scattering turbidimetry and endpoint scattering turbidimetry. The former is a dynamic detection method providing real-time scattering light intensity of antigen–antibody complexes, while the latter measures the reaction equilibrium as the total amount of antigen–antibody complexes [122]. Although traditionally employed for detecting more abundant CRP in serum due to its poor sensitivity, efforts are needed to improve sensitivity [123]. Particle-enhanced turbidimetric immunoassay emerged for determining analytes with relatively low concentrations, achieving quick detection on automatic biochemical analyzers. Latex particle-enhanced turbidity immunoassay is considered a reliable and easy-to-implement tool [124,125].

**Fig. 14.**
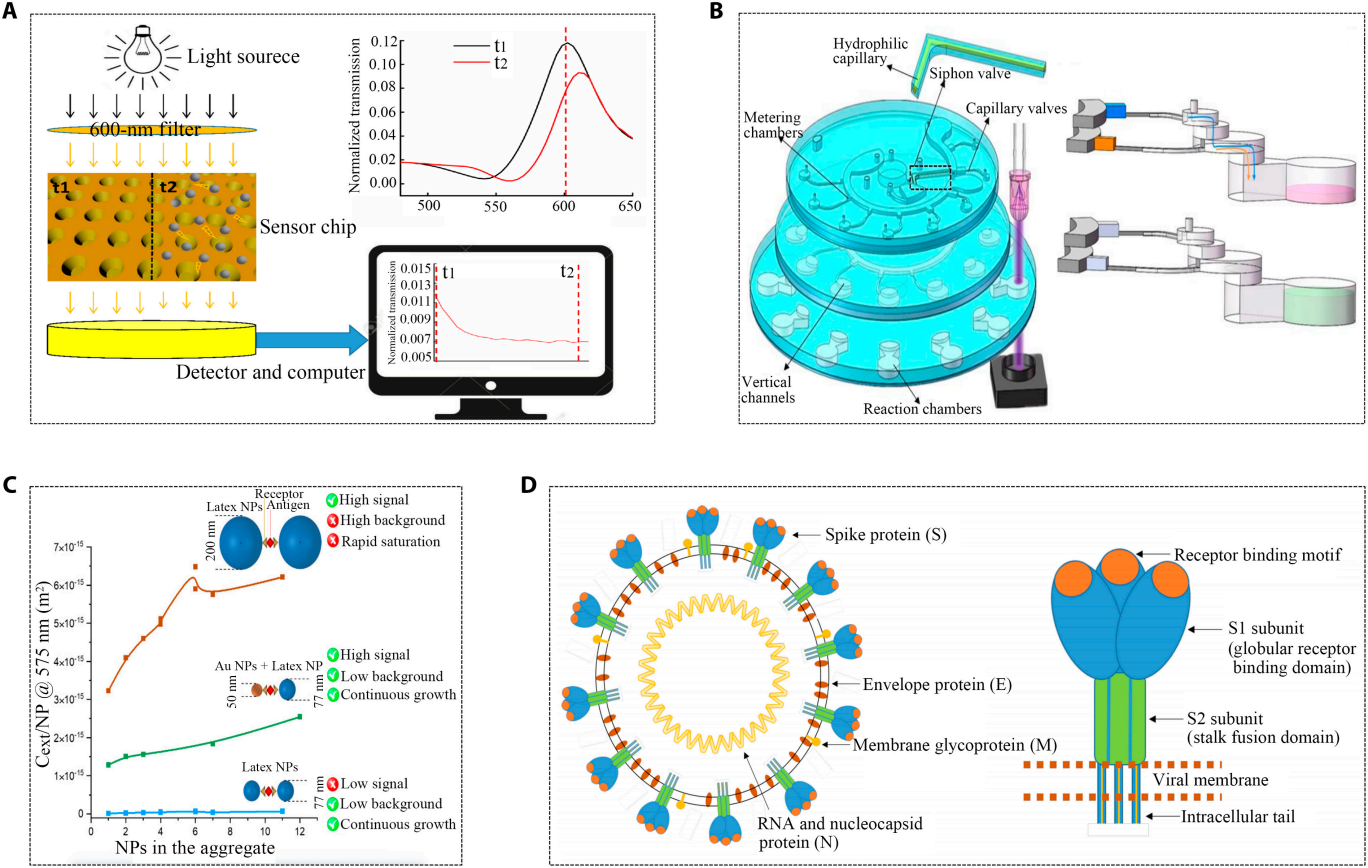
Applications of improved immunoturbidimetric assay for PCT detection. (A) The process of precipitation complex detection [128]. Copyright 2022 MDPI. (B) Schematic of the multi-layered disc and schematic of liquid flow between layers [132]. Copyright 2019 SPIE. (C) Plasmonic nanoparticles improve the sensitivity and dynamic range of immunoturbidimetric assays [129]. Copyright 2021 MDPI. (D) Particle-enhanced turbidity immunoassay based on viral nucleocapsids [126]. Copyright 2023 MDPI.

A standardized immunoassay reagent based on latex immunoturbidimetry for quantitative CRP detection has been developed, detecting CRP in a broad range of 0.2 to 320 mg/L and an accuracy exceeding 99%. Furthermore, a particle-enhanced turbidity immunoassay based on viral nucleocapsids has been designed to quantify immune responses to the virus, as depicted in Fig. 14D [126]. Another study introduced a microparticle turbidimetric reagent with high colloidal stability, utilizing immunopurified polyclonal antibodies. This approach allowed for lower antibody coverage on the particulate reagent, eliminating the drawbacks of non-specific agglutination in light scattering immunoassays and enhancing the colloidal stability of the reagent [127]. In addition to microparticles, there is also an ultrasensitive method called nanoplasmonic immunoturbidimetry assay. The increase in nanoparticle size induced a change in optical extinction or density value, which is measured at a certain wavelength with a universal microplate reader on the nanosensor chip. This improvement surpassed conventional immunoturbidity detection by more than 1,000 times. Not only can CRP be detected within a wide linear range of 1 to 500 ng/ml, the detection limit is reduced to 0.54 ng/ml, and the detection time only needs to be within 10 min. Because it is highly sensitive and effective in blood tests, this may become a novel technical system for detecting other biomarkers, including PCT (Fig. 14A and C) [128,129].

Microfluidic technology plays a crucial role in immune turbidity detection systems. Immunoturbidimetry, a quantitative analysis method based on antigen–antibody reactions, determines the concentration of antigens or antibodies in a sample by measuring the change in turbidity during the reaction. Microfluidic technology allows precise control of fluid flow in microchannels during immunoturbidimetric detection, facilitating rapid mixing, dilution, and sample separation. This enhances analysis speed and efficiency while reducing sample and reagent consumption, minimizing background interference, and improving detection accuracy. Additionally, microfluidic systems exhibit high integration and automation capabilities [130,131]. A research work proposed a new portable immunoturbidimetric detection system based on multi-layer centrifugal microfluidics, which precipitated blood cells in real time through centrifugation and quantitatively extracts purified plasma through a siphon valve. A real-time standard curve can be integrated on the chip. Since each test is individually calibrated, the interference from various environmental factors and manufacturing processes was greatly reduced, resulting in small error fluctuations. The experimental results are the same as those of the fully automatic biochemical analyzer. This research is expected to enable simultaneous detection of multiple specific proteins (Fig. 14B) [132]. Another research introduced a quantitative immune-agglutination analysis of a microvolume turbidity system integrating microfluidic technology. The system held promise in clinical diagnosis, particularly for small sample volume [133–135]. However, the consumables of microfluidic chips, such as test tubes and optical systems, can be expensive. A subsequent innovative turbidity determination system for quantitative immune-agglutination assays was constructed using off-the-shelf components. This system replaced disposable glass capillaries with inexpensive test tubes. The proximity extension assay (PEA), a homogeneous, dual-recognition immunoassay, demonstrated sensitivity, specificity, and convenience in detecting or quantifying one or more analytes in human plasma. The sensitivity of PCT in plasma samples was approximately 0.1 ng/ml [136,137]. Immune turbidimetry faced challenges, including excessive antibody requirements due to the hook effect between antigen and antibody, resulting in a narrow detection range. Additionally, immunoturbidimetry results were susceptible to sample quality issues, such as lipid blood, hemolysis, and jaundice interfering with absorbance signals.

## RIA for PCT Detection

RIA was developed to address these challenges by introducing a radioisotope-labeled antigen and unlabeled antigen to competitively bind antibodies [138,139]. Hence, the intensity of radiation is related to the amount of antigen measured. This quantitative method exhibited high sensitivity, usually reaching picomoles within a small number of samples. However, RIA uses radioactive isotopes that can be harmful to the human body. Furthermore, the technology required expensive equipment and consumables, limiting its application for PCT or other biomarkers.

**Fig. 15.**
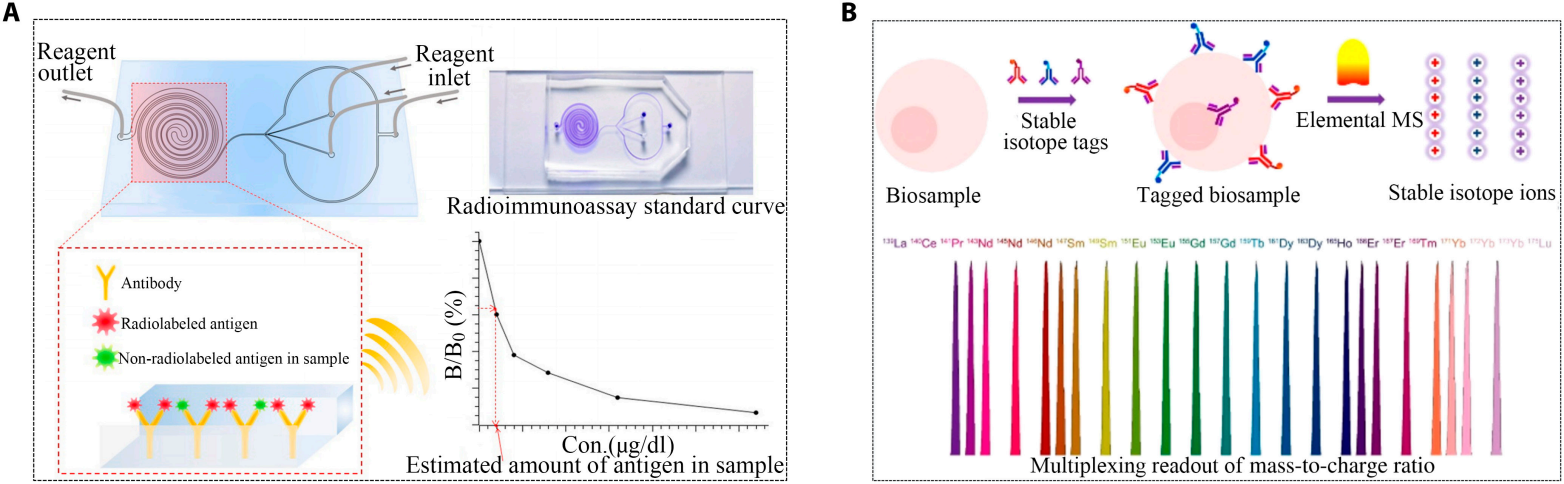
Applications of microfluidics and metal markers in radioimmunoassay for PCT detection. (A) Experimental scheme of μ-RIA [142]. Copyright 2021 Elsevier. (B) Schematic illustration of metal stable isotope atomic mass spectrometry analysis [143]. Copyright 2016 American Chemical Society.

Despite these limitations, RIA offered extremely high sensitivity and specificity. A study using RIA to measure calcitonin levels in rats demonstrated sufficient sensitivity with a range of 0.15 to 0.25 ng/ml [140]. Some studies indicated that liquid-phase RIA was more sensitive than solid-phase ELISA, IF, and endomysium antibodies (EMA) methods for specific autoimmune assays [141]. A microfluidic chip for RIA (μ-RIA) was designed for automated and high-throughput detection (Fig. 15A) [142]. However, due to poor specificity caused by polyclonal antibodies, it struggled to accurately distinguish free PCT, bound PCT, and PCT-related peptide precursors. In addition, the long turnaround time and the risks associated with radioactive substances limited the scope of RIA. To address the challenges posed by radioactive isotopes in RIA, metal stable isotope labeling may offer an alternative for biomolecule quantitation. With the advancement of atomic mass spectrometry measurement methods for metal stable isotopes, inductively coupled plasma mass spectrometry stood out for its excellent sensitivity, wide dynamic linear range, and multiple and accurate quantitative capabilities (Fig. 15B) [143]. However, the stability of metal stable isotope markers may be a concern, potentially leading to decomposition or inactivation and affecting detection results. In conclusion, representative innovative methods from each type were selected, and a table was created to summarize and compare them (Table).

**Table.** Comparison of PCT detection methods

Method	Innovation point	Limit of detection and detection range	References
CLIA	A novel CLIA kit and supporting instrument (AE-180)	0.0075 ng/ml, 0.01–110 ng/ml	[59]
	Desirable hyperbolic microfluidic chip	0.17 ng/ml, 0.4–12.8 ng/ml	[60]
	Active droplet-array microfluidics	0.044 ng/ml, 0.044–100 ng/ml	[61]
	SPE-C, drop detection	0.1 ng/ml, 0.5–1,000 ng/ml	[39]
	EMC-Au, on-chip detection	0.04 ng/ml, 0.1–20 ng/ml	[39]
	CeO_2_CuO-Au as a platform Au@Ag-Th as signal markers	0.17 pg/ml, 0.5 pg/ml–50 ng/ml	[40]
	Cu_2_S snowflakes as co-reaction accelerator	2.36 fg/ml	[41]
	MNP-based assay	0.03 ng/ml, 0.03–600 ng/ml	[38]
	PbS/Co_3_O_4_ as signal marker	0.02 pg/ml, 0.1 pg/ml–50 ng/ml	[45]
	Porous nanoarray BiVO_4_/Cu_x_S-signal amplification	17.3 fg/ml, 50–100 ng/ml	[46]
	BiVO_4_/GaON/CdS ternary nanocomposite	0.03 pg/ml, 0.1 pg/ml–50 ng/ml	[47]
	Antibodies immobilized on poly-3-hexylthiophene (P3HT)	2.2 pM	[48]
	g-C_3_N_4_ loaded onto MOF material	3.4 fg/ml, 0.014 pg/ml–40 ng/ml	[49]
	ECL immunosensor of CdS-MoS_2_ nanocomposite	33 fg/ml, 0.0001–10 ng/ml	[50]
	Label-free PEC immunosensor	0.14 pg/ml, 0.50 pg/ml–100 ng/ml	[51]
	3D carbonized wood-based integrated immunosensor	0.014 pg/ml, 0.05–90 pg/ml	[52]
	g-C_3_N_4_-CNT@Au as ECL donor	25.7 fg/ml, 0.0001–10 ng/ml	[54]
	Label-free ultrasensitive sensor of g-C_3_N_4_ NS	0.11 fg/ml, 0.15–11.7 fg/ml	[56]
IF	Quantitative immunofluorescence assay	0.1–100.0 ng/ml	[67]
	Micromotor-based fluorescence immunoassay	0.07 ng/ml, 0.5–150 ng/ml	[68]
	Nanocapsules as energy emitters and magnetic carbon dots	1–1,000 pg/ml	[70]
ELISA	Magnetic beads and enzyme antibodies labeling AuNPs	20 pg/ml, 0–8 ng/ml	[104]
	erPCT was produced for use as standard	25–1,000 ng/ml	[82]
	ALP as the marker enzyme and CuNCs as the tracer agent	7 pg/ml, 0.05–12 ng/ml	[84]
	WMC-assisted multi-channel detection platform	0.11 ng/ml, 0.4–12.8 ng/ml	[91]
GICT	Using the biotin–streptavidin system	0.25 ng/ml, 0.5–100 ng/ml	[101]
	CGE immune probe	0.025 ng/ml	[98]
	A gold-based paper sensor	0.1 ng/ml, 0.49–13.90 ng/ml	[97]
	Aggregated luminescent microspheres	3.8 pg/ml, 7.6–125 ng/ ml	[114]
	Highly luminescent quantum dot bead	0.0625 ng/ml, 0.0625–400 ng/ml	[115]
	Using GNSs, GNPNs and GNSTs as markers	0.1 ng/ml, 0.5–10 ng/ml	[111]
	Lanthanide chelate encapsulated polystyrene nanoparticles	0.05 ng/ml	[104]
	UCNPs@mSiO_2_ as a probe	0.5 ng/ml, 1–200 ng/ml	[105]
	Raman reporter molecules embedded in Au@Ag shell	0.042 ng/ml, 0–20 ng/ml	[108]
	Creating SERS signals on AuNPs	0.03 ng/ml	[109]
	ICA-SERS	8.017 pg/ml	[110]
	Dual detection function of protein and bacteria of MAFs	0.333 pg/ml	[112]
	Handheld FM-based PCT detection system	0.03 ng/ml, 0.04–50 ng/ml	[118]
TIA	Proximity extension assay	0.1 ng/ml	[137]
RIA	Radioimmunoassay to measure calcitonin levels	0.15 ng/ml, 0.15–0.25 ng/ml	[140]

GICT, immune colloidal gold technique; TIA, turbidimetric inhibition immunoassay.

## Clinical Application of PCT for Distinguishing Bacterial Infections

In clinical practice, assessing serum PCT concentration aids in identifying bacterial infections. Notably, a substantial increase in serum PCT is indicative of systemic reactions caused by bacterial infections, distinguishing them from other causes like autoimmune diseases, inflammation, and viral infections, where PCT levels typically remain low [144–146]. A typical study suggested an association between PCT concentrations and infective endocarditis, advocating for a lower PCT threshold to rule out endocarditis in routine clinical practice [147]. It is essential to highlight that PCT exhibited a rapid increase in severe bacterial infections within a few hours, offering an advantage over other inflammatory factors for early diagnosis [148]. Moreover, monitoring PCT changes allows for assessing the severity of bacterial infections and identifying the type of pathogenic microorganisms [149,150].

In addition, PCT can be used to distinguish between infectious and non-infectious causes of adult respiratory distress, and to distinguish between infectious necrosis and aseptic necrosis of pancreatitis [151]. In hematologic oncology, PCT also helps to make a definitive diagnosis of systemic infections caused by bacteria and fungi. PCT can reliably detect and assess sepsis infection even in chemotherapy patients. It can also be used to monitor infections after organ transplantation, especially those that arise during the rejection phase [152]. Many diseases are not specific in preterm infants and newborns, and PCT can also be used to diagnose and evaluate the treatment effect of these diseases [153].

## Clinical Application of PCT as a Diagnostic Indicator

PCT serves as a classic diagnostic indicator for sepsis [154,155]. Clinical analysis has demonstrated that a blood PCT concentration exceeding 2 ng/ml can diagnose sepsis caused by bacterial infection [156]. Additionally, PCT monitoring enabled distinguishing the infectious course of sepsis [157–159]. PCT plays a crucial role in guiding the rational use of antibiotics, aiming to reduce antibiotic misuse [157,160–162]. In clinical practice, it is recommended to discontinue antibiotics when the serum PCT is <0.25 ng/ml. If PCT content decreases by over 80% from the maximum value or falls into the range of 0.25 to 0.5 ng/ml, discontinuation of antibiotics is also advised. Conversely, when the PCT level exceeds 0.5 ng/ml and continues to rise, suggesting potential bacterial resistance, changing the antibiotic type is strongly recommended [155,158,163].

## Conclusions and Perspectives

The clinical application of PCT holds significant value, and various detection methods have emerged as powerful tools in clinical practice, especially with the growing interest in immunofluorescence and chemiluminescence. Immunoassay technology, renowned for its high specificity, sensitivity, and stability, has found widespread use in clinical diagnosis and biomedical research. In this review, we first introduced the fundamental concepts of PCT and PCT immunoassay to elucidate their basic principles and clinical importance. Then, we briefed several commonly used detection methods, including chemiluminescence, immunofluorescence, enzyme-linked immunoadsorption, latex-enhanced turbidimetry, colloidal gold immunochromatography, and RIA. We also summarized innovative implementation methods developed based on conventional approaches. From an alternative perspective, the research and development directions of immunoassay into magnetic bead immunoassay, Raman spectroscopy detection technology, nucleic acid immunoassay technology, and microfluidic immunoassay were categorized.

Despite distinct technological advancements and the application of new materials with superior properties, achieving early, sensitive detection, and dynamic monitoring during treatment remains challenging [164,165]. While remarkable developments have occurred in this field, challenges persist in achieving universal clinical applications, creating a gap between scientific experimental methods and practical techniques for solving real-world problems. Improvements are necessary in specificity, as actual samples like serum inevitably contain different biomarkers or inhibitors, leading to false-positive and false-negative results. Reagent stability and luminescence in tedious processes also require careful evaluation. Efficient procalcitonin tests (PCTS) have emerged as an alternative method, overcoming the time-consuming nature of blood culture and rapidly identifying bacterial infections [166,167]. PCT detection has matured with various modes, including non-background chromatography [168], surface-enhanced Raman spectroscopy with more obvious signal amplification [169], matrix-assisted laser desorption/ionization time-of-flight mass spectrometry [170], polymerase chain reaction nucleic acid amplification technology, isothermal rolling loop amplification technology at room temperature, etc. [171,172].

In short, despite these technical challenges, emerging technologies and functional materials will continue to contribute to technological innovation with highly sensitive and specific detection methods. Examples include signal reporting factors and real-time counting for single-molecule detection based on nanomaterials as carriers, catalytic compounds, and biosensors [99,173,174]. We anticipate that the scientific community will further promote its development, leading to new applications in the field of immunoassay.

### Ethics statement

The authors declare that human or animal ethics approval was not needed for this study.
